# Biomarkers for Transient Ischemic Attack: A Brief Perspective of Current Reports and Future Horizons

**DOI:** 10.3390/jcm11041046

**Published:** 2022-02-17

**Authors:** Masoud Nouri-Vaskeh, Neda Khalili, Alireza Sadighi, Yalda Yazdani, Ramin Zand

**Affiliations:** 1 Tropical and Communicable Diseases Research Centre, Iranshahr University of Medical Sciences, Iranshahr 7618815676, Iran; mnvaske@gmail.com; 2 Network of Immunity in Infection, Malignancy and Autoimmunity, Universal Scientific Education and Research Network, Tehran 1419733151, Iran; 3 School of Medicine, Tehran University of Medical Sciences, Tehran 1449614535, Iran; nedakhalili44@gmail.com; 4 Cancer Immunology Project (CIP), Universal Scientific Education and Research Network (USERN), Tehran 1419733151, Iran; 5 Neuroscience Institute, Geisinger Health System, Danville, PA 17822, USA; alireza.sadighi@yahoo.com; 6 Immunology Research Center, Tabriz University of Medical Sciences, Tabriz 5165665931, Iran; yaldayznn@gmail.com; 7 Neuroscience Institute, Pennsylvania State University, State College, PA 16801, USA

**Keywords:** biomarker, stroke, transient ischemic attack, TIA

## Abstract

Cerebrovascular disease is the leading cause of long-term disability in the world and the third-leading cause of death in the United States. The early diagnosis of transient ischemic attack (TIA) is of great importance for reducing the mortality and morbidity of cerebrovascular diseases. Patients with TIA have a high risk of early subsequent ischemic stroke and the development of permanent nervous system lesions. The diagnosis of TIA remains a clinical diagnosis that highly relies on the patient’s medical history assessment. There is a growing list of biomarkers associated with different components of the ischemic cascade in the brain. In this review, we take a closer look at the biomarkers of TIA and their validity with a focus on the more clinically important ones using recent evidence of their reliability for practical usage.

## 1. Introduction

Cerebrovascular disease is the leading cause of long-term disability and the second-leading cause of death worldwide [1]. Despite a slight decrease in ischemic stroke recurrence after a transient ischemic attack (TIA), it still carries a substantial risk of a subsequent ischemic stroke [2,3]. Therefore, the early recognition of TIA is of great importance. At the same time, the diagnosis of TIA remains a clinical diagnosis that highly relies on the patient’s medical history assessment [4]. However, the heterogeneous nature of TIA’s symptoms, symptom settlement at the time of first clinical encounter, and the absence of neuroimaging findings in the majority of patients makes the diagnosis even more challenging, especially for non-neurologists, and the risk of misdiagnosis is high [5,6,7].

A possible solution to the diagnostic difficulties in TIA could be an addition of serum or imaging biomarkers, or a combination of biomarkers that can reliably assist in the diagnosis of transient brain ischemia. Biomarkers could also provide valuable prognostic information [8]. Although there is a growing list of biomarkers associated with different components of the ischemic cascade in the brain, a practical biomarker should be sensitive to the early detection of ischemia and specific to the brain [9,10].

Due to the similarities in the pathological mechanisms and the ischemic cascade of TIA and ischemic stroke development, most of the available biomarkers can potentially be applied for the diagnosis of both diseases. Most of these biomarkers have a low predictive value for stroke in the hyperacute or reflect the severity of an established subacute or completed stroke. Therefore, introducing biomarkers for TIA can be more challenging since the degree of brain ischemia is much lower, and imaging and laboratory biomarkers are often closer to normal. In this systematic review, we evaluated the potential biomarkers and their validity for TIA diagnosis. We further discussed whether combining advanced statistics and machine learning models and a combination of clinical and paraclinical biomarkers could enhance prediction. We consider that a diagnostic panel for TIA should have a high positive and negative predictive value and should be able to detect the event up to several days after the transient symptoms.

## 2. Methods

A literature search was performed in databases including PubMed, MEDLINE, EMBASE, and Google Scholar to identify studies investigating potential diagnostic biomarkers in TIA published before 1 April 2021. The search strategy included keywords related to brain ischemia, biomarkers, and diagnostic studies. Since many studies had evaluated the role of biomarkers in diagnosing patients with either ischemic stroke or TIA, we searched for all studies of brain ischemia. The detailed search strategy can be found in Supplemental File S1. We also screened the reference lists of all included studies for any additional articles.

The primary search result was screened for duplicate and irrelevant papers by two independent reviewers considering the title and abstract of articles. Only English language studies were included and studies evaluating prognostic biomarkers were excluded from our systematic review. Full texts of selected articles were then reviewed by the same reviewers, and data were extracted from articles that were considered eligible for inclusion by consensus.

## 3. Results

The search protocol resulted in 8623 records from four literature databases (Figure S1). After the removal of duplicate records (N = 2402) and irrelevant articles based on their titles and abstracts (N = 6005), 216 discrete search results were screened. Of these potentially eligible studies, 132 articles were excluded after full-text review. More details are included in Figure S1.

### 3.1. Laboratory Biomarkers

#### 3.1.1. Biomarkers Specific to Neuronal Acute Ischemic Injury

The N-methyl-D-aspartate (NMDA) is a prototype agonist at the NMDA subtype of the ionotropic glutamate receptor. These receptors are prominently expressed in the hippocampal CA1, dentate gyrus, and striatum [11]. NMDA binds to NMDA receptors simulating the excitatory effects of glutamate, which plays an important role in learning and memory. The NMDA receptor has a tetramer structure and is composed of four subunits, including two glycine-binding NR1 and two glutamate-binding NR2 [12]. Anti-NMDA receptor antibodies develop in response to the release of peptide fragments resulting from NMDA receptor turnover during excitotoxicity, are recognized in the serum of stroke patients, and persisted for many months [12].

Following cerebral ischemia, the expression of the NR2 subunit is upregulated [13], in contrast to downregulation of the NR1 subunit [13,14]. In addition, based on existing evidence, cerebral ischemia and excitotoxicity induce the calpain-mediated cleavage of both NR2A and NR2B subunits [15]. Elevated plasma levels of the autoantibodies to NR2A/2B NMDA receptor subunits in plasma were reported in patients with TIAs and ischemic stroke, and the levels of NR2A/2B autoantibodies measured within 72 h differentiated ischemic stroke from intracerebral hemorrhage (ICH) [16]. Furthermore, NR2 may be a differentiation biomarker of TIA and ischemic stroke from stroke mimics [17].

In a study, serum samples of admitted patients with a TIA/stroke diagnosis were collected to measure autoantibodies to NR2A/2B in the following intervals after initial symptoms: 3, 6, 9, 12, 24, and 72 h. It was found that these autoantibodies could be used as a diagnostic tool after an ischemic event with the potential capability for the differentiation of ischemic lesions from ICH. They also described a TIA prognostic value for this test in routine clinical practice. The positive and negative predictive values were 93.0% and 96.0%, respectively [14]. The detection of autoantibodies to the NR2 subunits could also reveal a history of multiple strokes and worked as a predictive indicator for stroke [14,16]. In contrast, Dolmans et al. [18] found that the NR2 and NR2 antibodies had no added diagnostic value in suspected TIA with biomarker values below the detection range in 80.0% and 47.8% of patients, respectively. In the aforementioned study, NR2 levels were measured in serum, which had a 30% lower concentration that may explain these differences between studies, because the degeneration of NR2 by proteases affects its levels during a longer preanalytic phase. The NR2 and NR2 antibodies levels do not increase early after ischemia. Hence, the total time to sample storage is another important factor explaining different results.

DJ-1 protein, with encoding information on the PARK7 gene and a specificity for brain tissue, has several not clearly detected functions, mainly in Parkinson’s disease [19]. However, one of its functions is the protection of the brain cells from oxidative stress [20,21]. A postmortem analysis of cerebral spinal fluid (CSF) revealed an increase in DJ-1 protein expression compared to antemortem CSF analysis. Considering the fact that CSF changes could be a close reflection of cerebral tissue changes and the specific presence of DJ-1 protein in brain tissues, the idea of detection of DJ-1 protein serum changes in patients with cerebral ischemia could be of early diagnostic value for stroke, especially in advanced infarct age [19,22]. With a similar mechanism, postmortem CSF increases in nucleotide diphosphate kinase A (NDKA) are described to be useful in early diagnosis of stroke, within 3 h after index event [19]. The Tat-DJ-1 protein can be efficiently transduced in vitro and in vivo and markedly protects against oxidative-stress-induced cell death and ischemic insults [23].

Likewise, ubiquitin fusion degradation protein 1 (UFD1) is also reported to be increased in postmortem CSF analysis and has been put forward as a serum biomarker for the early diagnosis of TIA and ischemic stroke [24,25].

S100 calcium-binding protein B (S100B) and neuron-specific enolase (NSE), two cerebrum-specific proteins, have shown to be increased in patients with essential hypertension [26,27]. NSE is a dimeric isoenzyme of the glycolytic enzyme enolase and the neuron and neuroendocrine system’s cells are the main source of it [28]. High serum NSE concentrations have been associated with severe white matter lesions. Furthermore, hypertensive patients with higher serum NSE concentrations at baseline evaluation have had a higher chance of development of cerebrovascular accidents in the early course of patient follow-up. Overall, NSE serum concentration could be considered as an outcome prediction tool in hypertensive cases with cerebrovascular ischemic stroke.

S100B, a low-molecular-weight, glial-specific protein, mainly found in the cytoplasm and nucleus of astrocytes, is an important neuronal injury-related biomarker with at least 21 various subtypes that have been applied for the detection of cerebral ischemic lesions. For example, S100B serum level measurements have shown an incremental pattern following carotid endarterectomy in patients with poor outcomes and permanent ischemic lesions with positive confirmatory findings in diffusion-weighted imaging (DWI). Meanwhile, S-100B serum levels returned to the baseline within 24 h in patients with no cerebral ischemic lesions following carotid endarterectomy [29]. Serum astroglial protein S100B has been shown to have an association with clinical neurological deficit, infarct volume, functional disability, and functional disability after acute cerebrovascular events [30,31,32]. In another study conducted by Kumar et al., the S100 protein had an increased level from TIA to ischemic stroke, and then death [33], but S100B may only modestly differentiate TIA from acute ischemic stroke [34].

Tau is a protein contributed in the neuronal cytoskeleton structure [35]. Unmyelinated cortical axons are the major sites of tau expression [36]. The blood total tau levels are higher in acute ischemic stroke compared to patients with TIA, but tau is not capable of separating TIA from a stroke. However, it might have the potential to differentiate between ischemic events and stroke mimics [34].

#### 3.1.2. Biomarkers Specific to Glial Cell

Glial fibrillary-associated protein (GFAP), a monomeric intermediate filament protein, is generally found in astrocytes and, in lower amounts, in cerebral ependymal cells [10]. During cellular development, astrocytes can be distinguished from other glial cells by the presence of GFAP. Similar to NSE and protein S100B, any brain tissue damage can increase the CSF levels of GFAP [37]. Higher amounts of GFAP are detected in the CSF following ischemic brain tissue damage and hemorrhagic stroke [38]. Based on recent and strong supporting evidence, ICH and cerebral ischemic stroke can be differentiated by measurement of GFAP serum levels within 1–6 h [39,40] after stroke onset [41]. Early GFAP concentrations in ICH are more robust compared to ischemic stroke [42].

Although GFAP is a more sensitive biomarker for small cerebral ischemic injury and minor stroke in comparison with S100B [10], its delayed increase following mild ischemic injuries makes its clinical usage as a diagnostic tool limited [38,41,43]. One study has showed that transient ischemia stimulates GFAP gene expression in the gerbil neocortex, striatum, and hippocampus [44]. Nevertheless, evidence of the diagnostic value of GFAP is still preliminary, and to date, only speculations can be made about its use in patients with TIA. Further studies are needed to measure GFAP levels in the blood and/or CSF of patients with TIA.

#### 3.1.3. Biomarkers Related to Endothelial Injury

Nitric oxide (endothelium-derived relaxing factor), an endogenously biosynthesized gas with the chemical formula of NO, is produced in the blood vessels’ inner layer of endothelium. Furthermore, other than the basal formation of the NO gas, it is produced following endothelial exposure to various vasoactive agents in large vessels of the brain and arteriolar network. NO works as a smooth muscle relaxant and, therefore, dilates vessels and increases tissue blood supply [45]. The regular release of NO also plays a protective role against platelet and leukocyte aggregation in cerebral vessels’ endothelia. Various circulating vasculotoxic products and endothelial injuries can interrupt the normal function of the endothelium, and thus NO production. Patients with stroke were found to have vascular endothelial dysfunction associated with the pathophysiology of stroke and clinical severity of stroke [46]. Considering the importance of endothelial dysfunction in stroke pathophysiology, different endothelial-related metabolites were evaluated as potential biomarkers for cerebral ischemic lesions. An endogenous inhibitor of NO production, asymmetric dimethylarginine (ADMA), was associated with the atherosclerosis process. In a study by Wanby et al. [47], ADMA was found to be a powerful biomarker for TIA (odds ratio for highest versus lowest quartile 13.1; 95% CI: 2.9–58.6; *p*: 0.001); however, it was reported to be a feeble marker for acute stroke. The ADMA levels were increased compared with healthy controls (0.54 ± 0.05 vs. 0.50 ± 0.06 µmol/L).

Lipoprotein-associated phospholipase A2 (Lp-PLA2) is another recently described biomarker for cardiovascular and ischemic stroke. It is an enzyme that is produced following the activation of platelets and the inflammation process in atherosclerotic plaques [48]. It has been proposed that a higher mass or activity of the Lp-PLA2 enzyme is an indicator of the developed inflammatory process and oxidative stress. Therefore, Lp-PLA2 could be used as a biomarker to identify the beginning and progression of diseases such as ischemic stroke and cardiovascular disease, which have an atherosclerotic etiology. Tai and colleagues reported that Lp-PLA2 is not a useful biomarker for the diagnosis of acute brain ischemia after observing that no significant differences were observed in the Lp-PLA2 mass and activity levels between ischemic and non-ischemic patients [49]. In contrast, Kocak et al. found that the Lp-PLA2 enzyme activity was significantly lower in patients with acute ischemic stroke compared with the control group during the early stages of brain ischemia [50]. In a prospective study, increased Lp-PLA2 mass and activity were associated with higher chances of death/stroke composite outcome within 90 days following index TIA event [51]. These study findings were compatible with later study results for the consideration of a short-term prognostic value for Lp-PLA2 mass and activity in TIA and minor stroke patients [52,53].

Findings of the TIA cohort showed that anti-phosphatidylserine/prothrombin IgG antibodies are associated with clinical outcomes after TIA in patients without antiphospholipid antibody syndrome [54]. Likewise, lipoprotein-associated phospholipase A2 was found to be higher in large artery atherosclerosis in comparison to a non-large artery [52].

Peroxiredoxin 1 (PRDX1), an enzyme involved in oxidative stress, has the ability to identify cerebral infarction with a specificity of 86% and sensitivity of 53%, and could be a new biomarker in TIA diagnosis [55].

#### 3.1.4. Biomarkers Related to Coagulation

Fibrinogen, a 340 kDa glycoprotein with a circulatory concentration of 1.5–3 mg/mL, is a clotting factor [56,57] that can also be used as an inflammation biomarker [58]. The role of fibrinogen concentrations and the chance of ischemic heart disease development had been reported before [59,60,61]. Later reports provided some evidence regarding the relationship between the higher concentrations of plasma fibrinogen and increased risk of stroke [62,63,64], but a relationship between fibrinogen levels and stroke subtypes was not found [65]. On the other hand, more studies are necessary to clarify the association between fibrinogen concentration and ischemic stroke [66]. The risk of cerebral intraparenchymal hemorrhage was reported to increase in persons with high levels of plasma fibrinogen [56]. Patients with a history of TIA or ischemic stroke also have higher chances of recurrent ischemic stroke in the presence of high fibrinogen concentrations [67].

The 220 KDa multifunctional glycoprotein, von Willebrand factor (vWF), is produced in vascular endothelial cells and megakaryocytes and can be found in plasma, the extracellular matrix, intracytoplasmic Weibel Palade bodies (WPB), and alpha granules [68,69,70]. The fundamental role of vWF in the coagulation cascade for platelet adhesion and as a carrier of Factor VIII has been known for years. Animal model studies have shown that vWF has a regulatory action for cerebral vascular permeability closely related to stroke and inflammatory processes in the brain [71,72,73]. In a prospective study, patients with stroke and TIA represented an obvious elevation in vWF levels more prominently in patients with a recent symptomatic history of carotid stenosis [74]. vWF level measurements were performed at the following intervals: ≤4 weeks of TIA or ischemic stroke (baseline), ≥14 days, and then ≥90 days. vWF pro-peptide was not found to be a more sensitive marker for the detection of acute phase activation in endothelial cells. Patients with high levels of VWF showed more severe stroke, dismal clinical outcomes, and an increased chance of recurrent stroke [75,76]. However, in a post-acute phase study on children with ischemic stroke, vWF plasma levels in stroke patients were not meaningfully different from the control group and were excluded as a risk factor for ischemic stroke [77]. A genome-wide association study between the plasma levels of vWF and single-nucleotide polymorphisms (SNPs) has shown that the SNP, rs505922 (*p* = 2.32 × 10^−30^, variance explained = 6%) is strongly related to the vWF levels in the general studied population [76,78]. SNPs were also found to be located within or close to the ABO locus.

#### 3.1.5. MicroRNA and Cytoplasmic DNA Biomarkers

In an animal model study, MicroRNA (miRNA), especially obtained from the exosome of plasma and CSF samples, were found to be useful for the diagnosis of TIA [79]. The main idea of miRNAs evaluation originates from the findings of the gene expression changes in cerebral ischemic tissue following ischemic stress [80,81]. Simply, an early inflammatory response following ischemic injury in the cerebral tissue is the basis of the pathophysiologic mechanism for the consideration of tissue inflammation mediators as serum biomarkers for TIA.

In the study by Chinnery et al. [82] on the association between mitochondrial DNA and the risk of ischemic vascular events, the presence of mitochondrial DNA sub-haplogroup K was significantly less frequent in patients with TIA or stroke than in controls.

#### 3.1.6. Biomarkers Detected by Mass-Spectrometry-Based Proteomics

The basic principle of mass spectrometry (MS) is the measurement of molecular mass. In MS, after the ionization of chemical species, the ions are systematically arranged based on the mass-to-charge ratio. This technique plays an important role in the accurate study of proteins and the determination of their characteristics. There are several approaches for sample preparation in this method. The bottom-up method is a routine method that makes use of enzymatically produced peptides of complex protein mixtures that are subjected to liquid chromatography (LC) separation and two steps of mass spectrometry analysis. In the first MS acquisition, the masses of the intact peptides are determined, while the tandem MS (MS/MS) fragmentation process gives rise to cleavage products that break along peptide bonds, producing information on the identity and sequence of the protein as well as its modifications [83].

In a cohort study of two groups of patients with TIA and minor stroke [84], the serum sample collection of enrolled patients was performed within 48 h after symptom onset. The application of mass-spectroscopy-based proteomics for platelet basic protein serum concentrations revealed a significant increase in patients with ischemic lesions (more prominently TIA patients) compared to the control group. This study shows new evidence in support of the unbiased value of proteomics for the identification of novel serum biomarkers for early clinical diagnosis of TIA.

In a study by Fiedorowicz et al., levels of ceramide species and sphingosine-1-phosphate in the bloodstream were assessed by LC-MS/MS, based on coupling mass spectrometers together in a series to analyze complex mixtures. According to this study, sphingosine-1-phosphate/Cer-C24:1 ratio could possibly be useful in TIA diagnosis, especially in the long term after the index ischemic incidence [85].

#### 3.1.7. Inflammatory Cytokines and Other Biomarkers

The application of the microarray technique for the detection of gene expression characteristics paved the way to screen large numbers of genes involved in biological reactions and pathways. Therefore, this could be a useful method for the identification of different neurologic and related immune diseases [86,87,88]. Furthermore, this method could be a valuable way for the identification of molecular mechanisms of IS and the risk of the development of later complications [89]. Wang et al. reported that the serum levels of antibodies against metalloproteinase 1, chromobox homolog 1, and chromobox homolog 5 could be considered as potential biomarkers for TIA diagnosis [90].

Although there are partially limited data regarding the function of Chemokine receptor 7 (CCR7) in humans, it is worth further elaborating CCR7 function as a candidate outcome biomarker in ischemic cerebral events, considering its probable role in the process of ischemia-induced delayed neuronal death in an animal model study [91] and its upregulation in circulating leukocytes in patients with non-severe ischemic stroke [92]. CCR7 is a G protein-coupled chemokine receptor [92,93] that has been clearly detected on dendritic cells of the nervous system and T cells [94,95]. CCR7 was also found to be expressed in healthy brain astrocytes [96].

The induction of global TIA for 5 min in an animal model of gerbil, resulted in outstanding increases in both CCR7 immunoreactivity and protein levels of the pyramidal neurons in the CA1 region of the hippocampus [91]. Importantly, these changes could be time-dependent; for example, changes were detectable 5 days after ischemic-reperfusion injury in this animal model study.

The high-sensitive C-reactive (hsCRP) protein is associated with cardiovascular risk, predicts further ischemic events in patients with TIA, and was linked to an increased risk of recurrent cerebrovascular ischemic events [97,98]. Gong et al. [99] investigated the adult patients who presented with TIA or ischemic stroke with symptom onset within seven days and found that elevated hsCRP levels predicted poor outcomes in these patients, independently.

The predictive role of the soluble CD40 ligand was shown in cardiovascular events. Li et al. measured sCD40L levels in 3044 patients with TIA and acute minor stroke. According to this study, elevated sCD40L levels predict recurrent stroke in these patients, independently [100].

Arginine vasopressin (AVP), also called the antidiuretic hormone, is an important hormone in the human body with a key role in body fluid volume adjustment and vascular tone maintenance. The C-terminal part of the AVP precursor (CT-proAVP) is a glycopeptide with a 39-amino acid which is called copeptin [101]. Considering the very short half-life of AVP in the serum, copeptin is generally considered as a surrogate marker for AVP in clinical practice. Copeptin plasma concentrations and their alterations have been used as a diagnostic and prognostic biomarker in various diseases, for example: diabetes insipidus [102], heart failure, and myocardial infarction [103,104,105]. Currently, however, copeptin has found a new biomarker role in ischemic cerebrovascular disease. De Marchis et al. [106] report a prognostic value for copeptin in the prediction of stroke risk in TIA patients within 3 months after index ischemic injury. Furthermore, adjustment of the serum copeptin level with the ABCD2 score could improve this scoring system’s prognostic value for the prediction of stroke [107,108]. The importance of copeptin plasma level in TIA and ischemic stroke patients with cardioembolic etiology for the prediction of recurrent ischemic stroke and death in short-term and long-term follow-up series was also highlighted [107,108]. The diagnostic biomarkers related to body fluids, the level of evidence, and class of recommendation are shown in Table 1.

### 3.2. Clinical Diagnostic Models

Dawson [109] and the Diagnosis of TIA (DOT) [110] are the two main scoring systems. A set of Explicit Diagnostic Criteria for TIA (EDCT) [111], for differentiating between migraine and TIA, was also recently proposed. Although these scoring tools have not been adequately validated or proven to be useful in clinical practice, the DOT performed better in a direct comparison with Dawson in a cohort of 525 suspected TIA patients (c-statistic 0.89 versus 0.83) [110]. The EDCT was developed based on clinical practice and experience instead of statistical methods [111]. The criteria were originally developed with a focus on the differentiation between TIA and migraine with aura. Thus, the EDCT and its modified version needs further external validation with a wider range of non-vascular causes.

### 3.3. Biomarkers Related to Neuroimaging

In the realm of modern vascular neurology, the magnetic resonance diffusion-weighted imaging (DWI) modality has become the cornerstone of the diagnosis of ischemic cerebral pathologies in outpatient and inpatient settings.

Magnetic resonance imaging (MRI) seems to play a critical role in TIA diagnosis, as well as prognosis. About 75% of patients with TIA are found to have abnormalities on multimodal MRI, including DWI, perfusion-weighted imaging (PWI), and magnetic resonance angiogram (MRA) [112,113]. According to a prospective study by Nah et al., PWI is most useful for detecting ischemic lesions caused by large vessel diseases and cardioembolism in patients with negative DWI [114]. Additionally, DWI positivity is more commonly detected in the later phase after TIA compared to the early phase (1 to 12 h) [114]. About one-third of patients with suspected TIA have evidence of DWI positivity on initial MR imaging studies, characterized by a bright signal intensity change (hyperintensity). Studies have shown that TIA patients with a positive initial DWI are more likely to exhibit a subsequent infarct on follow-up imaging [115,116]. Patients without any detectable abnormalities on initial MR imaging modalities do not usually experience subsequent clinical events [112].

White matter hyperintensity (WMH) is a common finding of MR imaging in patients with ischemic stroke. Since WMH and TIA share similar risk factors, some have proposed that the assessment of pre-existing WMH lesion burden might help in improving the diagnostic certainty of TIA in DWI-negative patients [117,118]. In patients with recurrent TIA, leukoaraiosis is also associated with an increased risk of subsequent stroke [119]. Therefore, leukoaraiosis may provide both diagnostic and prognostic information in patients with suspected TIA.

In comparison to MRI, non-contrast computed tomography (CT) is less sensitive for detecting TIAs and minor strokes; acute ischemia is present in approximately 5% of these patients on non-contrast CT. Computed tomographic angiography (CTA), which is a non-invasive gold standard vascular imaging technique in cerebrovascular diseases [120], is helpful in predicting the risk of stroke recurrence. For instance, the CATCH study demonstrated that the presence of asymptomatic intracranial or extracranial vessel occlusion or stenosis of ≥50% on CTA or acute ischemia on non-contrast CT could be predictive of stroke recurrence 90 days after the initial event [121].

In a pre-clinical mouse model study [122], the combination of ultra-sensitive molecular magnetic resonance imaging using antibody-based microparticles of iron oxide targeting P-selectin in the endothelium of brain vessels after the induction of experimental transient ischemic attack helped differentiate TIA from two main TIA mimics: epilepsy and migraine. In this study, MRI patterns related to the upregulation of P-selectin molecules were found to be visible after 6 h of ischemia induction, reached a peak of about 24 h, and had a substantial reduction 48 h after experimental TIA induction. Furthermore, some recent studies evaluated the role of pH imaging as a new technique in the detection of moderate-to-severe metabolic injury as a complementary step for stroke MRI for the classification of heterogeneous tissue response following brain ischemic lesions [123,124,125]. However, there is no reliable report regarding the usefulness of the application of this technique following TIA, and we still need more evidence to emerge in this regard.

In a quantitative study of blood–brain barrier (BBB) integrity, the application of dynamic contrast-enhanced MRI for mapping BBB dysfunction among patients with transient neurological deficits showed that these patients had a significantly higher BBB dysfunction compared with healthy controls. This study is considered to be the first report on the promising predictive value of BBB dysfunction localization and its extent in patients with transient ischemic attack/minor stroke. BBB dysfunction mapping could be a valuable biomarker tool in the detection of subtle brain ischemia and a predictive biomarker for risk assessment and stroke prevention in patients with TIA [126].

### 3.4. Electroencephalogram (EEG) and TIA Diagnosis

The application of functional brain measures such as EEG may help in understanding the mechanism of TIA. Elevated alpha and beta activity in acute EEG may be associated with TIA and be helpful in the elucidation of TIA mechanism [127]. Bentes et al. reported focal slow wave activity as the most common abnormality in the early EEG of these patients [128].

### 3.5. Retinal Findings Using Fundus Photography

The investigation of retinal findings using fundus photography holds promise for the future of TIA research. For instance, a study found that focal and general arteriolar narrowing could differentiate patients with TIA from those presenting to the emergency department with focal neurologic deficits [129]. In the FOTO-TIA study, Bruce and colleagues showed that retinal microvascular findings detected with non-mydriatic fundus photography had the potential to differentiate patients with TIA or stroke from their mimics [130]. In addition, they found that smaller arteriovenous ratios (AVR < 0.63) and central retinal artery equivalents (CRAE < 160 mm), larger central retinal vein equivalents (CRVE > 237 mm), and the presence of cotton wool spots, retinal hemorrhages, or focal arteriolar narrowing on fundus photographs were associated with DWI positivity [131]. However, this study failed to demonstrate a prognostic role for fundus photography, as microvascular retinal changes were not predictive of 90-day risk of stroke in patients with suspected TIA [132]. These results suggest that retinal examination could be an accessible surrogate to identify and stratify patients with TIA via noninvasive and rapid methods; therefore, the inclusion of fundus photography in acute stroke protocols deserves further evaluation.

### 3.6. Combination of Biomarker Panels and techniques

In a study by Zhan et al. [133], a panel of 34 genes was investigated for differentiation between patients with TIA and persons with vascular risk factors without symptomatic cardiovascular disease. The time of sampling was an average of 35 h after symptom onset. A prediction analysis showed a panel of 34 genes that discriminated TIA from controls with 100% sensitivity and 100% specificity. Another study on a panel of 26 genes was conducted to find the differentiation between TIA or ischemic stroke and controls [133]. The time of sampling was 72 h after symptom onset. Prediction analysis showed that this panel discriminated TIA or ischemic stroke from controls with 89% sensitivity and 89% specificity. A panel of 26 genes reported by Jickling et al. [134] may distinguish TIA and stroke from controls with 89% specificity and sensitivity.

Findings derived from a large-scale translational study using multiple reaction monitoring MS showed that a panel of 16 proteins, of which 9 (including L-selectin, insulin-like growth factor-binding protein 3, coagulation factor X, serum paraoxonase/lactonase 3, thrombospondin-1, hyaluronan-binding protein 2, heparin cofactor 2, apolipoprotein B-100 and von Willebrand factor) were significant univariate predictors of TIA and could help to distinguish TIA from mimics [135].

**Table 1:** Biomarkers for TIA diagnosis in body fluids.

Biomarker Type	Biomarker	Status	COR	LOE	Serum/Plasma/CSF	Stroke and TIA	Stroke/TIA and Mimics	Reference(s)
	NR Peptide	Downregulated	IIb	C-LD	Serum	No	Yes	[13,14]
	NR2 Peptide	Upregulated	IIb	C-LD	Plasma	No	Yes	[17]
	Autoantibodies to NR2A/2B NMDA receptor subunits	Increased	IIb	C-LD	Serum	No	No	[16]
	Ubiquitin fusion degradation protein 1 (UFD1)	Increased	IIa	C-LD	Plasma/Serum	No	Yes	[24]
	S100 calcium-binding protein B (S100B)	Increased	IIb	C-LD	Serum	No	No	[29,136]
	Neuron specific enolase (NSE)	Increased	IIb	C-LD	-	-	-	[26]
	Heart-fatty acid binding protein (H-FABP)	Increased	IIb	C-LD	Plasma	No	Yes	[137]
	Myelin basic protein (MBP)	No change	IIb	C-LD	CSF	No	Yes	[138]
	Neurofilament Light Chain (NFL)	Decreased	IIb	C-LD	Serum	Yes	Yes (stroke only, not TIA)	[139]
	DJ-1 (PARK7)	Increased	IIb	C-LD	Plasma	No	Yes	[25]
	NDKA	Increased	IIb	C-LD	Plasma	No	Yes	[25]
	T-tau	Increased	IIb	C-LD	Serum	Yes	No	[34]
Endothelial-Related Biomarkers	Asymmetric dimethylarginine (ADMA)	Increased	IIa	C-LD	Plasma	No	Yes	[47,140]
	lipoprotein-associated phospholipase A2	Increased	IIb	C-LD	-	-	-	[52,53]
	Glutathione S-Transferase-π	Increased	IIb	C-LD	Serum	No	No	[141]
	Fibrinopeptide A	Increased	IIb	C-LD	Serum	No	No	[142]
	von Willebrand factor (vWF)	Increased	IIb	C-LD	Plasma	No	No	[74]
	Antibodies against metalloproteinase 1, chromobox homolog 1, and chromobox homolog 5	Increased	IIB	C-LD	Serum	No	Yes	[90]
	Platelet basic protein (PBP)	Increased	IIb	C-LD	Serum	No	Yes	[84]
	Chemokine receptor 7 (CCR7)	Increased	NA*	NA*	-	-	-	[91]
mRNA	Panel of 34 genes	-	IIb	C-LD	Serum	No	No	[133]
	Panel of 26 genes	-	IIb	C-LD	Serum	No	No	[134]

## 4. Discussion

In our systematic review, we have identified different biomarkers (Table 1) using various techniques. There are variations in TIA presentation, etiologies, as well as the timing and setting of medical evaluation (outpatient vs. inpatient) [6,7,143]. Therefore, a diagnostic panel for TIA should be highly positive and have negative predictive values, regardless of the etiology, able to label the diagnosis in an acute phase as well as up to several days after transient symptoms, preferably available in outpatient practice and inexpensive. Furthermore, given the high risk of stroke in the first few days after a true TIA event and a high risk of TIA misdiagnosis, the results should be available promptly. Our results suggest that none of the evaluated biomarkers meets the above criteria and can be used as an accurate diagnostic tool based on the current evidence. Although there might be several promising biomarkers, many of the respective studies were limited due to small sample size, lack of validation, and lack of diverse control and consideration of all TIA mimics. A candidate panel should be able to differentiate between TIA and TIA mimics with other CNS pathologies as well as healthy subjects regardless of etiologies. Further studies on biomarkers panels and techniques may help to develop TIA diagnosis. Many factors—including cost, timing, accessibility, and reliability—should be taken into the consideration.

For now, TIA remains a clinical diagnosis that highly relies on the patient’s medical history assessment; however, several imaging and laboratory biomarkers can further narrow down the diagnosis and exclude TIA mimics.

DWI-MR imaging studies of the brain have become the main clinical tool for the diagnosis of acute cerebral ischemic lesions [144]. However, the application of MR imaging modalities has some limitations in specific clinical settings. For example, it is not accessible in all hospitals, especially after hours; it is associated with high cost; and not every patient can have it (pacemakers, agitated patients, metal implants, etc.). It should be highlighted that the reliability of MRI is undermined by the lack of its technical ability to diagnose patients with TIA. Nonetheless, DWI-MR is very sensitive to the diagnosis of stroke and can differentiate stroke from stroke mimics. None of the current candidate biomarkers are reliable enough to replace DW-MRI for ischemia. We believe that imaging will remain as the most helpful diagnostic and prognostic tool among patients with cerebral ischemia; however, imaging can be combined with novel clinical and serum biomarkers to further characterize the event, measure the severity of ischemia and differentiate or define a transient event.

Furthermore, given the similarity of inflammatory and atherosclerotic pathophysiology and reactions that result in ischemic accidents, not only in the cerebrovascular lesions but also in cardiovascular ischemic injuries, any research targeting the detection and study of biomolecules as a biomarker should take this similarity into the account. In other words, as long as we are not able to find highly specific biomarkers for each of these clinically different entities, any potential biomarker might not be specific and reliable enough to make clinicians the needles of MR studies for accurate diagnosis. The incorporation of new analytical chemistry techniques for a better understanding of molecules involved in ischemic lesions’ pathophysiology, for example mass spectrometry and chromatography, might help to open new horizons [84].

It has been known that different cerebral tissue cells have various tolerances to ischemia [145]. Furthermore, considering the cell diversity in the brain compared to the cardiovascular system, we could hypothesize that the detection of structure-specific biomarkers of the brain could help in the creation of a schematic view of the possible anatomical location of ischemic lesions. We need to know the correct picture of cell level reactions and metabolomics involved in ischemic processes, the minimum amount of time needed for the detection of tissue-specific ischemic injury, and the minimum size of tissue injury that could produce a measurable level of biomarkers. Moreover, the plasma/serum half-life of possible biomarkers could help for understanding the time-frame of tissue injury when minutes matter in deciding on a proper diagnosis and intervention.

In addition, many studies used a healthy control and ignored the fact that patients with stroke or TIA-like symptoms can have several central nervous systems (CNS)-related alternative diagnoses. A helpful study should include stroke and TIA mimic patients as the control group.

Finally, biomarkers could have a prognostic value which might be more valuable among patients with TIA given the higher chance of short-term recurrent ischemic attack outcomes. Among this group, neuroimaging, including noninvasive angiograms, has significant value. New advances in Big Data and machine learning methods can also introduce new horizons in disease prediction and outcome [146].

## 5. Conclusions

In conclusion, none of the evaluated biomarkers can be recommended for TIA diagnosis. Clinical diagnostic models, biomarkers related to neuroimaging, and a panel of genes are better than a single biomarker. However, we believe more studies related to diagnostic panels that combine imaging, serum, clinical and other novel biomarkers should be considered in the future.

## Data Availability

Not applicable.

## Supplementary Materials

The following supporting information can be downloaded at: https://www.mdpi.com/article/10.3390/jcm11041046/s1, Figure S1: Flow diagram of the systematic review; Supplemental File S1: Search strategy.
