# Supplementary material for: Biomarkers for Transient Ischemic Attack: A Brief Perspective of Current Reports and Future Horizons
Source: J Clin Med. 2022 Feb 17;11(4):1046. doi: 10.3390/jcm11041046 (PMC8877275; doi:10.3390/jcm11041046)
Supplement: Supplementary file 1 [file jcm-11-01046-s001.zip › Supplemental File S1.pdf]

Supplemental File S1. Search strategy

Search strategy:

1. biological markers[MeSH Terms] OR biomarker*[Title/Abstract] OR marker*[Title/Abstract] OR blood test*[Title/Abstract] OR laboratory test*[Title/Abstract] OR imaging biomarker*[Title/Abstract]

2. biomarker*[Title/Abstract] AND (perfusion computed tomogra*[Title/Abstract] OR PCT[Title/Abstract] OR CT perfusion[Title/Abstract] OR perfusion-weighted MRI[Title/Abstract] OR perfusion MRI[Title/Abstract] OR magnetic resonance perfusion[Title/Abstract] OR MR perfusion[Title/Abstract] OR computed tomography angiogra*[Title/Abstract] OR CT angiogra*[Title/Abstract] OR CTA[Title/Abstract] OR magnetic resonance angiography[MeSH Terms] OR magnetic resonance angiogra*[Title/Abstract] OR MR angiogra*[Title/Abstract] OR MRA[Title/Abstract])

3. 1 OR 2

4. sensitive*[Title/Abstract] OR specific*[Title/Abstract] OR diagnos*[Title/Abstract] OR likelihood functions[MeSH Terms] OR likelihood ratio[Title/Abstract] OR sens*[Title/Abstract] OR spec*[Title/Abstract] OR roc curve[MeSH Terms] OR area under curve[MeSH Terms]

5. ischemic attack, transient[MeSH Terms] OR brain ischemia[MeSH Terms] OR TIA[Title/Abstract] OR TIAs[Title/Abstract] OR (transient isch*[Title/Abstract] AND attack*[Title/Abstract]) OR brain isch*[Title/Abstract] OR cerebral isch*[Title/Abstract]

6. 3 AND 4 AND 5
