# Supplementary figures and images for: Biomarkers for Transient Ischemic Attack: A Brief Perspective of Current Reports and Future Horizons
Source: J Clin Med. 2022 Feb 17;11(4):1046. doi: 10.3390/jcm11041046 (PMC8877275; doi:10.3390/jcm11041046)

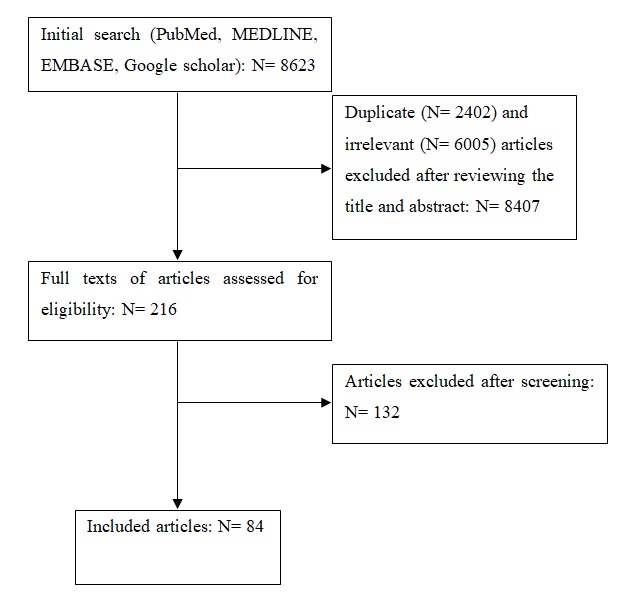

Supplement: Supplementary file 1 [file jcm-11-01046-s001.zip › Figure S1.jpg]
